# Dietary Intake among Children Attending Childcare Centers: Impact of the New CACFP Meal Guidelines

**DOI:** 10.3390/nu14163394

**Published:** 2022-08-18

**Authors:** Jayna M. Dave, Tzuan A. Chen, Maha Almohamad, Sonia Cotto-Moreno

**Affiliations:** 1 USDA/ARS Children’s Nutrition Research Center, Department of Pediatrics, Baylor College of Medicine, 1100 Bates Street, Houston, TX 77030, USA; 2 Psychological, Health, and Learning Sciences Department, University of Houston, Houston, TX 77204, USA; 3 Health Research Institute, University of Houston, Houston, TX 77204, USA; 4 Department of Epidemiology, Human Genetics and Environmental Sciences, School of Public Health, The University of Texas Health Science Center at Houston (UTHealth), Houston, TX 77030, USA; 5 Dietitians Balance Health, San Antonio, TX 78254, USA

**Keywords:** Child and Adult Care Food Program, meal patterns, early childhood nutrition, childcare, food policy

## Abstract

Through the Healthy, Hunger-Free Kids Act of 2010, USDA (US Department of Agriculture) made the first major changes in the CACFP (Child and Adult Care Food Program) meal and snack menu patterns. Childcare centers that serve low-income families qualify to participate and receive reimbursement for meals and snacks served. The purpose of this study was to assess what changes in children’s dietary behaviors occurred as a result of the new CACFP meal pattern requirements. This study evaluated these changes at childcare centers operating in Houston and San Antonio, Texas, USA, areas enrolled in the CACFP, pre- (Spring 2016) and post-implementation (Fall 2016–Spring 2017) of the new meal patterns. Dietary intake was assessed via observations of children, 3–5 years old, conducted at breakfast, lunch, and snack times. Results showed improvements in adjusted means of several nutrients and food groups consumption, post-implementation of new CACFP meal guidelines compared to baseline, specifically intake of whole grains, milk, and juice. Additional studies are needed to confirm the impact of the revised CACFP meal patterns along with strategies to assist providers in meeting the new standards to increase the access to and intake of items in accordance with the CACFP meal pattern requirements in childcare settings.

## 1. Introduction

Early childhood development sets the stage for school readiness, educational attainment, and adult health and well-being [1]. Development in early childhood is associated with a higher demand for nutrients and energy to support physical and cognitive development, emotional and social development, and rapid recovery following infection [2,3,4,5]. The interplay of nutritional status and child development cannot be overemphasized as it can impact their immediate and future health [6]. Children’s dietary patterns and nutrition habits are formed in early childhood which serves as a foundation for future preferences and intake [7,8]. In 2019, 62% of children, ages 5 years and younger, attended center-based programs such as day-care centers, prekindergarten programs, Head Start, or other early childhood programs, where they spent an average of 33 h per week [9,10]. Efforts to improve nutrition and influence dietary habits in childcare settings may be particularly effective [11,12,13]. It is recommended that childcare facilities provide half to two-thirds of the recommended dietary allowances for children in full-day childcare [14], placing a great deal of responsibility on the provider to ensure nutritionally adequate, healthful food.

Childcare centers that serve low-income families may participate in the US Department of Agriculture’s (USDA) Child and Adult Care Food Program (CACFP) and receive reimbursement for meals and snacks served [15]. CACFP has been serving children in childcare settings since 1968. Childcare centers may be approved to claim up to two reimbursable meals (breakfast, lunch, or supper) and one snack, or two snacks and one meal, to each eligible participant each day [15]. CACFP reimburses the centers at free, reduced-price, or paid rates for these meals and snacks if the program meal standards are met. Eligibility to receive CACFP benefits is based on household income [15]; a child is eligible for free or reduced-price meals if their gross monthly household income is at or below 130% (i.e., at or below 1.3 times the current federal poverty level) or 185% (i.e., between 1.3 and 1.85 times the current federal poverty level) of the US federal poverty level guidelines, respectively [16]. In 2019, CACFP served over 4.4 million children with snacks and meals, totaling about 2.1 billion meals; approximately 75% of all meals were served in childcare centers [17,18].

Through the Healthy, Hunger-Free Kids Act of 2010, the USDA made the first major changes in the CACFP meal and snack menu patterns since the program began in 1968 [19]. The changes to the meal patterns were based on the Dietary Guidelines for Americans (DGA) [20], science-based recommendations made by the National Academy of Medicine (NAM) (formerly the Institute of Medicine of the National Academies) [19], cost and practical considerations, and stakeholder’s input. These improvements to the meals served in the CACFP were expected to safeguard the health of young children. The changes require centers participating in the CACFP to serve more whole grains and a greater variety of vegetables and fruit and reduce the amount of added sugar and saturated fat in snacks and meals. Moreover, as of October 2011, only non-fat and low-fat (1%) unflavored milks were to be served to children 2 years and older attending childcare centers receiving CACFP reimbursements [21]. There are also optional best practices that will enable childcare centers to further improve meal quality [22]. These build on the CACFP meal patterns and highlight areas where centers may take additional steps to further improve the nutritional quality of the meals they serve and reflect recommendations from the DGA [20] and the NAM [19]. Updated CACFP nutrition standards were implemented in October 2017.

The current CACFP meal patterns include up to four meal components: fluid milk, fruits/vegetables, grain/bread, and meat/meat alternates, depending on meal occasion [22]. Minimum required amounts of the meal components and serving sizes differ by age group. For children ages 3–5 years, breakfast includes three meal components: one serving each of milk (6 ounces), fruit or vegetable (1/2 cup), and grain or bread (1/2 serving). Lunch and supper meal patterns include four meal components: 1 serving each of milk (6 ounces), grain or bread (1/2 serving), meat/meat alternate (1.5 ounces), and 2 different servings of fruit or vegetable or a combination of fruit and vegetable (1/2 cup total). Snacks include two of the four meal components (milk (4 ounces), fruit or vegetable (1/2 cup), grain or bread (1/2 serving), or meat/meat alternate (1/2 ounce)). Facilities can choose to serve two meals and a snack, or two snacks and a meal each day [22].

One of NAM’s report recommendations was for research identifying how the new meal requirements would change children’s program-related dietary intakes [19]. Thus, the purpose of this study is to assess what changes in children’s dietary behaviors occurred as a result of the new CACFP meal pattern requirements.

## 2. Materials and Methods

This study is an evaluation of a natural experiment to assess the changes in dietary intakes of children at childcare centers operating in Houston and San Antonio, Texas, USA, areas enrolled in the CACFP, pre- (Spring 2016) and post-implementation (Fall 2016–Spring 2017) of the new CACFP meal patterns. A natural experiment is when exposure to the event or intervention of interest has not been manipulated by the researcher [23].

### 2.1. Sample

A convenience sample of 20 licensed childcare sites operating in the two Texas cities and enrolled in CACFP, participated in the study. Note that the licensing requirements in Texas align with the CACFP. These centers were invited to participate because they were not participating in other projects that required menu changes at the time of recruitment. Although the mandate of implementing the new CACFP guidelines was not until October 2017, all 20 participating sites decided to make the required changes in Summer/Fall 2016. Out of the twenty sites, seven sites were considered small (serving < 31 children), eight sites were considered medium-sized (serving between 31–69 children), and five sites were considered large (serving ≥ 70 children). This study was approved by the Institutional Review Board at Baylor College of Medicine (H-35784). The director of each center agreed to participate in the study. Observations were conducted anonymously; therefore, individualized parental consent was not required. Each site received an honorarium of USD 500 for their participation.

### 2.2. Staff Training

Six research staff attended a 3 h training session to review procedures and learn how to conduct meal observations and use the meal observation form using the protocol developed by Ball and colleagues [24]. The observation protocol developed by Ball and colleagues has been used in previous research in childcare centers [24,25]. Each observer conducted 2 to 4 practice observations, with the previously trained research coordinator also recording consumption. Acceptable interrater reliability was obtained (>90%). The research coordinator conducted quality control checks with each data collector once a month for quality control.

### 2.3. Data Collection

Dietary intake at CACFP sites (centers and homes) was assessed via observations of children, 3–5 years old, conducted at breakfast, lunch, and snack times. Trained observers observed each center 6–8 times during pre- and post-implementation periods. Observations were conducted on different weekdays in each site; 3–4 children were observed per site per meal.

The research coordinator mapped each childcare center, identified the seating arrangements, and established the daily data collection rotation for each site. The data collectors conducted the observations based on this rotation. Each data collector observed three to four children at breakfast, lunch, and/or snack and recorded all items and the amount eaten on a pre-printed meal checklist. For each item, the amount eaten was recorded using the quarter waste method (0, 1/4, 1/2, 3/4, all), which has a high inter-rater and inter-method reliability [26]. The data collector observed from a distance and recorded the age and gender of the observed child. If asked what they are doing, the observers were instructed to say that they were just interested in seeing how the children were enjoying their lunch. Meal observation data were entered into the Nutrition Data System (University of Minnesota, MN, USA) by trained dietitians to obtain nutrient and food group intake.

### 2.4. Statistical Analysis

Separate analyses were conducted for meal data. Sample comparisons of characteristics of interest based on time of data collection (i.e., pre- and post-implementation) were performed using chi-square tests. Spring and fall data were unmatched at the participant level; therefore, analysis of covariance (ANCOVA) was conducted to examine the time effect which was considered a between-group fixed factor. ANCOVA was carried out separately for each outcome of interest to compare the mean amounts of nutrients and food groups consumed adjusted for child’s gender, city, center type, and center size. The results presented are adjusted means, standard errors for calories, nutrients, and food components for children pre- and post-implementation of the new CACFP meal guidelines. All analyses were conducted using SAS 9.4 (SAS Institute, Inc., Cary, NC, USA) [27] and level of significance was designated at *p* < 0.05.

## 3. Results

A total of 1855 valid observations were conducted at pre-implementation—523 at breakfast, 658 at lunch, and 674 at snack time. At post-implementation, a total of 1736 observations were conducted—515 at breakfast, 616 at lunch, and 605 at snack time. Table 1 shows participant characteristics by collection time and meals. For breakfast, compared to post-implementation, participants at pre-implementation were significantly more likely to be sampled from San Antonio (*p* = 0.006) and more likely to be from Head Start (*p* = 0.019) and from centers of small size (*p* = 0.007). For lunch, compared to post-implementation, participants at pre-implementation were significantly more likely to be from centers of small size (*p* = 0.039). For snacks, compared to post-implementation, participants at pre-implementation were significantly more likely to be sampled from Head Start (*p* = 0.013).

Compared with the breakfast of the children sampled at pre-implementation, children at post-implementation had significantly less consumption of added sugars (*p* = 0.001), refined grains (*p* < 0.001), and serving of juice (*p* < 0.001). Additionally, a significantly greater intake of calcium (*p* < 0.001), vitamin C (*p* = 0.019), whole grains (*p* < 0.001), and total milk (*p* < 0.001) were found at post-implementation of guidelines compared to the pre-implementation (Table 2).

At lunch, children at post-implementation consumed significantly lower amounts of added sugars (*p* < 0.001), serving of juice (*p* = 0.026), starchy vegetables (*p* = 0.036), total protein foods (*p* = 0.044), total all vegetables (*p* = 0.002), and snack chips (*p* = 0.002) compared with children at pre-implementation. Additionally, children at post-implementation consumed significantly greater amounts of calcium (*p* = 0.039), vitamin C (*p* = 0.009), total grains (*p* = 0.030), whole grains (*p* = 0.0001), and total milk (*p* = 0.0007) compared to pre-implementation (Table 3).

With regards to the snack pattern components, children at post-implementation consumed significantly higher amounts of whole grains (*p* = 0.001), total protein foods (*p* = 0.036), and snack chips (*p* < 0.001) compared to pre-implementation. However, a significantly lower amount of total milk was found for children at post-implementation compared to pre-implementation (*p* = 0.026) (Table 4).

## 4. Discussion

In response to the report of the National Academy of Medicine’s recommendations calling for research identifying how the new CACFP meal requirements would change children’s program-related dietary intakes, we sought to assess these changes in children’s dietary behaviors. To the best of our knowledge, this is one of the first few studies to explore the impact of the new CACFP meal pattern requirements on dietary behavior changes among these children. Overall, results were encouraging, as discussed below, and showed modest improvements in food and nutrient consumption post-implementation of the new meal guidelines.

Intakes of whole grain foods increased during breakfast, lunch, and snacks, while refined grain intakes decreased at breakfast. Total grain intake increased at lunch. These findings are similar to a study conducted in Boston, MA, USA which found an increase in whole grain intake and a decrease in refined grains intake during lunch in 3–5-year-old children [28]. However, the majority of grain intake was still from refined grains.

Unlike another study that found an increase in fruit intake [28], there was no change in fruit intake among the study sample across the three meals post-implementation. However, there was a decrease in vegetable intake during lunch. Several studies have found similar results for vegetable intake with young children consuming vegetables infrequently [28,29,30,31]. Increasing fruit and vegetable intake among young children is important since many do not currently consume adequate servings of fruits and vegetables [32,33], and those who do may be less likely to become overweight or obese [34] and have a decrease in all-cause mortality across the lifespan [35].

Juice intake decreased at breakfast and lunch but did not change at snacks. This was probably because the juice was limited to once per day and was only served at snack post-implementation of the new CACFP meal patterns [36]. No juice intake at breakfast and lunch may also be the reason for overall decreases in sugar intake during those meals in addition to no desserts or flavored milk being served at the sites [36].

Post-implementation, intake of total protein foods decreased during lunch but slightly increased at snack time. In general, it is important to increase the protein intake among young children since lean proteins, particularly vegetable proteins, may be protective against excess body weight [37,38]. Thus, protein is an important target of CACFP meal patterns, considering prior evidence that children in these settings do not consume adequate amounts of lean proteins [39,40,41].

Intake of milk, and therefore calcium, increased during breakfast, lunch, and snacks post-implementation of the new CACFP meal patterns. Although more milk was consumed at all three meals, intake still fell short of the recommendations (6 ounces at breakfast and lunch each, and 4 ounces at snack when served). Milk is a critical source of high-quality protein, calcium, vitamins A and D, and total caloric intake among young children [42]. All the participating sites served only nonfat or 1% milk; no flavored milk was offered [36]. Additionally, recent studies have showed that most children consumed plain, unflavored, reduced-fat (2%), or low-fat (1%) milk in childcare centers [43,44,45].

Finally, the percent calories from fat and saturated fats significantly increased at breakfast post-implementation of the new meal patterns. In fact, percent calories from fat and saturated fat were higher than recommended for all the three meals. This may be attributed to the fact that the sites did not meet the new CACFP meal pattern guidelines and best practices of providing only lower-fat meat and meat alternates, limiting processed meats to <1 serving/week, and serving only natural cheeses, choosing low-fat or reduced-fat cheeses, and limiting pre-fried foods to ≤1 serving/week [36]. In fact, regular beef and full-fat cheese products were commonly served [36]. Although certain fats (such as omega-3 fats) are important for brain growth, and neural and cognitive development in young children, overconsumption can lead to overweight and obesity and metabolic health risks later in life [46].

In general, USDA’s goal can be met through the implementation of the new CACFP meal pattern requirements to improve children’s nutrient and food intake. This is just one study observing a small sample of childcare centers in Texas showing the positive impact of the revised meal requirements. However, there is still more research needed to leverage CACFP to improve young children’s overall diet. Support and assistance may be needed for the providers, including increased reimbursements for the costs of providing healthy foods, to better meet the goals of the revised meal patterns. Researchers should work to identify and efficiently convey the barriers to full implementation of the new meal guidelines and best practices and how providers can be better supported.

### 4.1. Limitations

Several limitations should be noted. First, the study was conducted in twenty childcare sites in the Houston and San Antonio areas of Texas; thus, the findings might not be representative or generalizable to the entire state of Texas and the US. Since we evaluated a natural experiment, there was a non-random selection of the sites. Additionally, children were not tracked from pre- to post-implementation of the new CACFP guidelines and thus we could not account for the potential impact of picky eating or food neophobia. Further, the study lacked a comparison group that did not experience the CACFP meal pattern revisions. Without the comparison group, we cannot be sure that any changes observed were solely due to CACFP meal pattern changes, although we monitored and found no other policies and programs related to nutrition were accessible to the providers during the time of the study. Another limitation is related to the assessment of the amount of food by observation, not by weighing the foods left on the tray (plate waste). However, the observation method used has been found to be valid and reliable [47]. As such, the observational data are considered more accurate and have reduced social desirability bias than self-reported data [48], but may not accurately represent normal practice or account for in-depth variability of nutritional intake behaviors [49]. Moreover, due to limited funding, we were not able to assess the longer-term implications of the policy. Despite this, findings from this study are encouraging. Regular monitoring of children’s food consumption at childcare centers is needed to assess whether the new meal patterns improve intake at these centers.

### 4.2. Implications for Future Research

The new CACFP meal guidelines are designed to make significant improvements to the nutritional quality of meals and snacks served through CACFP. Future research should:

- Include observational and prospective studies to confirm these results and provide wider and more representative conclusions of the new standards.
- Assess the impacts of the revised meal patterns in the longer term, making use of longer follow-up periods.
- Compare the meal consumption of pre-school aged children throughout the day (at and outside of childcare centers) which would provide a better understanding of their intake and areas for improvement.
- Continue developing and evaluating strategies to increase the accessibility and intake of items in accordance with the CACFP meal pattern requirements in childcare settings.

Additionally, providers and childcare centers should model healthful eating practices by implementing the new CACFP meal pattern requirements and encouraging children to accept a variety of nutrient-dense food groups. This is one of the first studies to evaluate the impact of the new CACFP meal guidelines on children’s dietary intake, employing more robust data collection methods. Overall, the study findings contribute to the positive changes that come from new and revised formal nutrition policies.

## 5. Conclusions

Results in this study showed modest improvements in adjusted means of several nutrients and food group consumption across the three meal types, breakfast, lunch, and snacks post-implementation of new CACFP meal guidelines compared to baseline. In this sample of childcare centers, children’s dietary intake improved on some of the domains targeted by the revised CACFP meal patterns—specifically whole grains, milk, and juice. Improvements in other domains, however, were not observed. While additional studies in other areas and childcare settings are needed to further evaluate the impact of the revised CACFP meal patterns, these results also suggest that providers may need more assistance in meeting the new standards.

## Figures and Tables

**Table 1:** Participant characteristics.

	Breakfast ^a^		Lunch ^b^		Snack ^c^	
	Pre	Post	Pre	Post	Pre	Post
	*n* (%)	*n* (%)	*n* (%)	*n* (%)	*n* (%)	*n* (%)
Gender						
Boy	228 (43.59)	238 (46.21)	303 (46.05)	262 (42.53)	308 (45.70)	287 (47.44)
Girl	295 (56.41)	277 (53.79)	355 (53.95)	354 (57.47)	366 (54.30)	318 (52.56)
City ^a^**						
Houston	187 (35.76)	227 (44.08)	340 (51.67)	328 (53.25)	367 (54.45)	320 (52.89)
San Antonio	336 (64.24)	288 (55.92)	318 (48.33)	288 (46.75)	307 (45.55)	285 (47.11)
Site Type ^a^*^c^*						
Home	7 (1.34)	19 (3.78)	28 (4.26)	19 (3.18)	17 (2.52)	31 (5.28)
Day care center	26 (4.97)	34 (6.76)	136 (20.67)	142 (23.75)	134 (19.88)	133 (22.66)
Head Start	490 (93.69)	450 (89.46)	494 (75.08)	437 (73.08)	523 (77.60)	423 (72.06)
Site Size ^a^**^b^*						
Small < 31	131 (25.05)	88 (17.09)	225 (34.19)	171 (27.76)	199 (29.57)	161 (26.61)
Medium 31–69	232 (44.36)	248 (48.16)	249 (37.84)	247 (40.10)	284 (42.20)	248 (40.99)
Large 70+	160 (30.59)	179 (34.76)	184 (27.96)	198 (32.14)	190 (28.23)	196 (32.40)

Note. *: significant distribution difference between time-point and variable of interest; * *p* < 0.05; ** *p* < 0.01; ^a^: Breakfast; ^b^: Lunch; ^c^: Snack.

**Table 2:** Adjusted means of nutrients and food groups consumption at breakfast.

	Pre (*n* = 523)		Post (*n* = 515)	
Nutrients	Mean	SE	Mean	SE
Energy (calories)	210.635	9.895	217.615	9.259
% Calories from fat	38.240	4.011	39.291	3.812
% Calories from SFA	14.553	1.552	14.535	1.473
Sodium (mg)	280.254	18.507	271.499	17.318
Calcium (mg) ***	190.982	12.962	234.243	12.129
Vitamin A (IU)	1459.875	78.979	1516.287	81.442
Vitamin C (mg) *	11.592	0.855	14.101	0.633
Added sugars (g) **	7.864	0.811	6.324	0.759
**Food Groups**				
Whole grains (ounce equivalents) ***	0.164	0.042	0.266	0.040
Refined grains (ounce equivalents) ***	0.720	0.061	0.582	0.057
Total grains (ounce equivalents)	0.884	0.063	0.848	0.059
Fruit (serving)	0.620	0.063	0.662	0.059
Juice (serving) ***	0.016	0.024	−0.073	0.023
Starchy vegetables (serving)	−0.001	0.001	−0.003	0.001
All vegetables (serving)	−0.008	0.008	−0.002	0.008
Total milk (fluid ounces) ***	3.425	0.285	4.299	0.267
Total protein foods (serving)	0.262	0.041	0.256	0.039
Snack chips	0.000	0.000	0.000	0.000
Snack bars	0.151	0.022	0.142	0.020

Note. * *p* < 0.05; ** *p* < 0.01; *** *p* < 0.001; SE = standard error; SFA = saturated fat.

**Table 3:** Adjusted means of nutrients and food groups consumption at lunch.

	Pre (*n* = 658)		Post (*n* = 616)	
Nutrients	Mean	SE	Mean	SE
Energy (calories)	312.505	9.332	306.052	9.229
% Calories from fat	47.451	1.785	44.082	1.741
% Calories from SFA	17.046	0.656	15.671	0.646
Sodium (mg)	582.319	22.701	553.505	22.451
Calcium (mg) *	246.584	12.169	266.819	12.030
Total vitamin A activity (IU)	1406.783	143.588	1459.996	141.953
Vitamin C (ascorbic acid) (mg) **	9.565	1.454	12.811	1.153
Added sugars (by total sugars) (g) ***	5.293	0.342	3.847	0.328
**Food Groups**				
Whole grains (ounce equivalents) ***	0.086	0.041	0.214	0.040
Refined grains (ounce equivalents)	0.821	0.038	0.773	0.037
Total grains (ounce equivalents) *	0.907	0.046	0.987	0.046
Fruit (serving)	0.516	0.030	0.537	0.030
Juice (serving) *	0.001	0.002	−0.003	0.002
Starchy vegetables (serving) *	0.161	0.018	0.130	0.018
All vegetables (serving) **	0.630	0.037	0.539	0.037
Total milk (fluid ounce) ***	4.708	0.244	5.375	0.241
Total protein foods (serving) *	1.116	0.062	1.016	0.061
Snack chips **	0.011	0.013	−0.022	0.013
Snack bars	0.002	0.003	−0.000	0.003

Note. * *p* < 0.05; ** *p* < 0.01; *** *p* < 0.001; SE = standard error; SFA = saturated fat.

**Table 4:** Adjusted means of nutrients and food groups consumption at snack.

	Pre (*n* = 674)		Post (*n* = 605)	
Nutrients	Mean	SE	Mean	SE
Energy (calories)	153.134	5.094	150.974	4.752
% Calories from fat	42.583	2.625	42.304	2.591
% Calories from SFA	12.995	0.884	11.809	0.862
Sodium (mg)	183.379	8.202	174.448	7.652
Calcium (mg) *	155.707	6.962	149.253	6.505
Total vitamin A activity (IU)	524.411	70.561	464.556	65.830
Vitamin C (ascorbic acid) (mg) **	1.783	0.982	2.226	0.917
Added sugars (by total sugars) (g) ***	4.610	0.320	4.933	0.299
**Food Groups**				
Whole grains (ounce equivalents) ***	0.159	0.026	0.228	0.025
Refined grains (ounce equivalents)	0.647	0.035	0.608	0.032
Total grains (ounce equivalents) *	0.806	0.039	0.836	0.046
Fruit (serving)	0.129	0.024	0.112	0.022
Juice (serving) *	0.165	0.035	0.125	0.033
Starchy vegetables (serving) *	0.000	0.000	0.000	0.000
All vegetables (serving) **	0.029	0.012	0.025	0.011
Total milk (fluid ounce) ***	2.569	0.161	2.286	0.149
Total protein foods (serving) *	0.059	0.026	0.103	0.024
Snack chips **	−0.039	0.015	0.035	0.014
Snack bars	0.472	0.030	0.452	0.028

Note. * *p* < 0.05; ** *p* < 0.01; *** *p* < 0.001; SE = standard error; SFA = saturated fat.

## Data Availability

Data are available from the corresponding author on reasonable request.
